# Efficacy of High Resolution Magnetic Resonance Imaging in Preoperative Local Staging of Rectal Cancer

**DOI:** 10.4274/Mirt.43153

**Published:** 2013-08-01

**Authors:** Aysun Uçar, Funda Obuz, Selman Sökmen, Cem Terzi, Özgül Sağol, Sülen Sarıoğlu, Mehmet Füzün

**Affiliations:** 1 Dokuz Eylül University, Department of Radiology, İzmir, Turkey; 2 Dokuz Eylül University, Department of General Surgery, İzmir, Turkey; 3 Dokuz Eylül University, Department of Pathology, İzmir, Turkey

**Keywords:** Rectum, rectum cancer, magnetic resonance imaging, tumor staging

## Abstract

**Objective:** To assess the efficacy of high-resolution magnetic resonance imaging (HRMRI) for preoperative local staging in patients with rectal cancer who did not receive preoperative radiochemotherapy.

**Methods:** In this retrospective study, 30 patients with biopsy proved primary rectal cancer were evaluated by HRMRI. Two observers independently scored the tumour and lymph node stages, and circumferential resection margin (CRM) involvement. The sensitivity, specificity, the negative predictive value and the positive predictive value of HRMRI findings were calculated within the 95% confidence interval. The area under the curve was measured for each result. Agreement between two observers was assessed by means of the Kappa test.

**Results:** In T staging the accuracy rate of HRMRI was 47-67%, overstaging was 10-21%, and understaging was 13-43%. In the prediction of extramural invasion with HRMRI, the sensitivity was 79-89%, the specificity was 72-100%, the PPV was 85-100%, the NPV was 73-86%, and the area under the curve was 0.81-0.89. In the prediction of lymph node metastasis, the sensitivity was 58-58%, the specificity was 50-55%, the PPV was 43-46%, and the NPV was 64-66%. The area under the curve was 0.54-0.57. When the cut off value was selected as 1 mm, the sensitivity of HRMRI was 38-42%, the specificity was 73-82%, the PPV was 33-42%, and NPV was 79-81% in the prediction of the CRM involvement. The correlation between the two observers was moderate for tumour staging, substantial for lymph node staging and predicting of CRM involvement.

**Conclusion:** Preoperative HRMRI provides good predictive data for extramural invasion but poor prediction of lymph node status and CRM involvement.

**Conflict of interest:**None declared.

## INTRODUCTION

Rectal cancer, defined as a tumour with its lower edge within 15 cm from the anal verge, accounts for about a third of all colorectal malignancies. Management is particularly challenging technically for the surgeon and local recurrence within the pelvis is a common result of treatment failure. The introduction of the concept of total mesorectal excision (TME) has resulted in a decline in local recurrence rates (1). There is evidence that preoperative radiotherapy may further reduce the rate of local tumour recurrence (2,3). The benefits of radiotherapy seem most marked in patients with T3, T4 or node positive disease. Involvement of the circumferential resection margin (CRM) by tumour is believed to be the main cause of local recurrence after rectal cancer surgery (1,4,5). The clinical challenge is to identify preoperatively the cohort of rectal cancer patients who are at high risk of local tumour recurrence. Preoperative radiotherapy could then be applied selectively to this subgroup. Hence, accurate preoperative staging is of paramount importance. Recent publications have suggested that detailed rectal anatomy can be demonstrated using thin section magnetic resonance (MR) imaging with a pelvic phased-array coil (5,6,7). This technique permits accurate T-stage determination and determination of the tumour involving surgical resection margins. The ability of thin section MR imaging in identifying the mesorectal fascia has been demonstrated, but there is conflicting evidence regarding the overall accuracy of MR imaging in staging rectal cancer (7,8,9,10,11,12,13). The purpose of our study was to assess the overall diagnostic accuracy of HRMRI for preoperative T and N staging and prediction of the CRM involvement in patients with rectal cancer who had not received preoperative radiochemotherapy.

## MATERIALS AND METHODS

**Patients**

Institutional Review Board approval was obtained for this study. Between April 2003 and January 2008, 203 consecutive patients with biopsy-proved rectal cancer were staged preoperatively using HRMRI. Thirty of these patients (18 males and 12 females; mean age, 65.9 (range, 25–80) years) who had undergone surgical resection and had not been given preoperative radiochemotherapy were included in this retrospective study. Rectal tumours were defined as tumours within 15 cm of the anal margin. 

**MR Imaging Techniques**

All patients underwent HRMRI with a 1.5 T system (Philips Gyroscan Intera Release 8, Eindhoven, Netherlands). A four-element pelvic phased-array surface coil was used. Patients were examined in the supine position after applying an antispasmodic agent (Buscopan). Patients did not undergo rectal air insufflations, nor did they receive bowel preparation or intravenous contrast. Sagittal fast spin-echo T2-weighted (TR/TE:3500–4000/70-85, section thickness 3 mm, intersection gap 0.8 mm, matrix 256 x 512, number of signals acquired 6, field of view 22 cm) images were obtained. These images were used to plan fast spin-echo T2-weighted para-axial images perpendicular to the long axis of the tumour. These images were obtained using an 18-cm field of view. The other parameters were the same as sagittal images. The last sequence was fast spin-echo T2-weighted para-coronal images parallel to the long axis of the tumour (field of view 22 cm). All images were reviewed using a high definition monitor and Easy-Vision software supplied by Philips Medical System.

**Evaluation of Images**

The patient’s staging was done according to the TNM five classification. Briefly, T2 tumours involve the muscularis propria, T3 tumours extend to the perirectal fat and T4 tumours directly invade other organs or structures, and /or perforate visceral peritoneum. N0 status refers to absence of nodal disease. Stage N1 denotes the presence of one to three malignant nodes and stage N2 denotes the presence of four or more malignant nodes. (please insert a reference)We used a size criterion of 5 mm maximum short axis nodal diameter for discriminating between benign and malignant nodes.(please insert a reference) For the prediction of the CRM, the observers assessed the HRMRI scans for the shortest distance from the outermost part of the tumour to the adjacent mesorectal fascia at the level of the maximum depth of penetration through the bowel wall. The distance was measured on the axial images with an Easy Vision Workstation (Philips Medical Systems). If a cancer was staged T1 or T2, the observer measured the shortest distance from the bowel wall at the level and site of the tumour to the adjacent mesorectal fascia. When an extramural tumour deposit or suspected lymph node was located nearer to the mesorectal fascia than to the primary tumour, this was used for measuring the closest distance to the fascia. If the distance between tumour or involved lymph node and mesorectal fascia was ≤1 mm, CRM was defined as involved. When the distance was more than 1 mm, CRM was defined as uninvolved. When the tumour invaded another organ (stage T4), the mesorectal resection plane was involved. 

**Surgery**

All patients underwent standard mesorectal excision in the pre-sacral plane. This technique involved sharp dissection of the rectum and its surrounding fat within an intact mesorectal fascia. The inferior hypogastric nerves were preserved. Anteriorly, the specimen included intact Denonvillier’s fascia and peritoneal reflection.

**Pathology**

The extent of local tumour staging was assessed according to the TNM system. The CRM involvement was calculated. 

**Statistical Analysis**

The results of HRMRI analyzed by the two observers were correlated with pathologic staging (pT and pN staging). Agreement between radiologic staging of the tumour, local lymph nodes, and CRM involvement with pathologic reporting and agreement between the two observers were assessed by means of the kappa statistics. For extramural invasion, metastatic lymph node involvement and CRM involvement, the sensitivities, specificities, the negative predictive values and the positive predictive values of HRMRI were calculated within the 95% confidence interval. The observer performances were examined by analysis of ROC curves. The area under the curve was used to indicate the overall performance of HRMRI. 

Statistical analyses were performed with SPSS 15.0.

## RESULTS

Depending on the localization, rectal carcinoma was in the distal rectum in 12 patients, in the middle rectum in 13 patients, in the superior rectum in 5 patients.

According to histopathologic staging of the 30 patients, 7 (23.3%) had pT1, 4 (13.3%) had pT2, 12 (40%) had pT3, and 7 (23.3%) had pT4 tumour (Table 1). Eighteen patients (60%) were classified as N0, nine patients (30%) were classified as N1 and three patients (10%) were classified as N2.

In T staging, the accuracy rate of HRMRI was 47-67%, overstaging was 10-21%, and understaging was 13-43% according to both observers. In the prediction of extramural invasion with HRMRI, the sensitivity was 79-89%, the specificity was 72-100%, the PPV was 85-100%, and the NPV was 73-86%. The area under the curve was 0.81-0.89.In predicting lymph node metastasis, the sensitivity was 58-58%, the specificity was 50-55%, the PPV was 43-46%, and the NPV was 64-66%. The area under the curve was 0.54-0.57. 

When the cut off value was selected as 1 mm, the sensitivity of HRMRI was 38-42%, the specificity was 73-82%, the PPV was 33-42%, and NPV was 79-81% in the prediction of the CRM involvement. The area under the curve was 0.63-0.65. 

Statistically, there was good correlation between pathologic and radiologic evaluation of extramural invasion (k=0.73 according to the first observer, k=0.63 according to the second observer). There was poor correlation between pathology and radiology in the prediction of lymph node metastases (k=0.07 according to the first observer, k=0.13 according to the second observer). There was fair correlation between pathologic and radiologic reporting of CRM involvement (k=0.25 according to the first observer, k=0.20 according to the second observer). However, the correlation between the two observers was moderate for tumour staging, substantial for lymph node staging and prediction of CRM involvement. Results are summarised in Table 2, 3, 4.

## DISCUSSION

Colorectal cancer is a major health problem and its’ incidence is increasing (14). One-third of all colorectal cancers occur in the rectosigmoid or rectal region. Rectal cancer carries a poor prognosis because of the risk both for metastases and for local recurrences. After curative resection, local recurrence rates of rectal cancer can vary from 3% up to 32% (15). Local recurrence is usually nonresectable and is associated with unpleasant symptoms that are difficult to palliate. Although postoperative radiotherapy allows selection of patients on the basis of histopathological risk factors, it seems to have little impact on survival (16). In contrast, preoperative (neoadjuvant) radiochemotherapy has been shown to reduce the rates of local recurrence and to improve survival (2,3). The major weakness of this strategy is its toxicity (17). There is a need for accurate preoperative staging to allow patient selection for such treatment.

**T Staging**

Transrectal MRI using an endorectal coil can generate images with good spatial resolution due to its high signal-to-noise ratio. This provides more accurate information about wall penetration than conventional MRI. However, as in the case of transrectal ultrasound, its use is limited by the necessity for specialised, dedicated equipment, poor patient acceptability and limited access to the tumour in patients with high or stenosing lesions. The assessment of the mesorectal fascia is also hampered by its limited field of view (5,9,18,19,20).

With the introduction of new imaging sequences and thin section technique (HRMRI), Brown et al. was able to demonstrate 100% accuracy and complete interobserver agreement in the staging of 25 primary rectal lesions (7). This initial high accuracy and reproducibility however was not confirmed. Blomqvist et al., using a 1.5 T scanner and pelvic phased-array coil, reported that the accuracy of predicting a T3 lesion was 78%, with a sensitivity of 86% and specificity of 65% (9). Using a similar technique, Beets-Tan et al. showed overall accuracy rates of 67-83% in the prediction of the tumour stage. There was also substantial interobserver variability in this study. Most of the staging failures occurred in differentiating T2 from early T3 lesions (8). According to Brown et al. only tumours with rounded or nodular advancing margin out with the muscularis propria should be regarded as T3 lesions (7). However, Beets-Tan et al. had difficulties in differentiating peritumoral inflammation or fibrosis from early tumour infiltration and concluded that tumours with fine strandings or spiculations in the adjacent perirectal fat should be classified as T3 lesions (8). Possible reasons for these differences may be due to the differing patterns of use of preoperative radiotherapy; all patients in the study of Brown et al. had received short course radiotherapy one week before resection (7). A recent meta-analysis including 21 studies performed with MRI using phase-array coil demonstrated 87% sensitivity, 75% specificity and 20.4 diagnostic odds ratio (DOR) for T staging (21).

**Figure 1:**
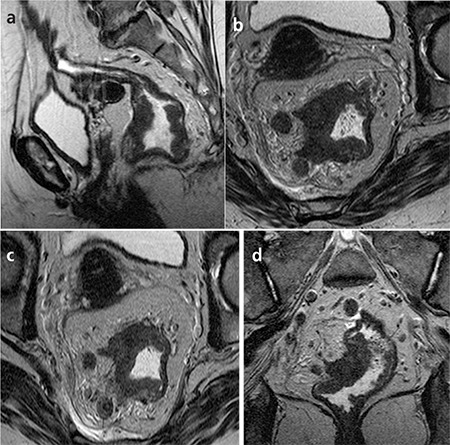
54-year-old woman with pT4N1 rectal cancer. Two observers staged the tumor as T3N2 in the preoperative MR imaging. a- T2-weighted sagittal MR image shows the tumor. b- T2-weighted para-axial MR image shows the tumor with nodularextramural invasion and perirectal lymph node metastasis. c- On T2-weighted para-axial MR image, the distance betweeninvolved lymph node and mesorectal fascia was more than 1 mm andCRM was defined as uninvolved. But histopathologically CRM wasdefined as involved.d- T2-weighted para-coronal MR image shows the tumor and lymph nodes.

In our study depending on the tumour localization, 12 patients had lower, 13 had middle and 5 had superior rectal cancer. Our overall T-staging accuracy was 47-67%. In the prediction of extramural invasion with HRMRI, the sensitivity was 79-89%, the specificity was 72-100%, the PPV was 85-100%, the NPV was 73-86%, and the area under the curve was 0.81-0.89. There was good correlation between pathologic and radiologic evaluation of extramural invasion. Two of the 30 patients were evaluated wrongly as T3 lesions. As mesorectum is thinner in lower rectum than superior, staging failures in that localization is a common problem. In our study, 8 patients having lower rectum cancer were staged wrongly: four of them were overstaged and the rest were understaged. The overstaged 4 tumours were predicted as T3 lesions because of inappropriate visualizing of the muscularis propria. The first observer understaged 5 patients as T3 that 4 of were invaded in visceral peritonium (Figure 1).

**N Staging**

The preoperative assessment of regional lymph node status forms part of the overall staging of any rectal tumour. Nodal status remains a critical prognostic factor in patients with rectal cancer. Accurate prediction of nodal status may thus influence preoperative treatment strategies. The application of a size criterion of 5 mm maximum short axis nodal diameter for discriminating between benign and malignant nodes has a moderate sensitivity and specificity for the detection of nodal metastases (5,22,23). In a meta-analysis of imaging studies used for the staging of rectal cancer, it was found that there were no significant differences among endorectal sonography, CT, and MRI in nodal staging. The sensitivity and specificity of endorectal sonography, CT, and MRI for detecting lymph node metastasis were 67% and 78%, 55% and 74%, and 66% and 76% respectively (10). In a comparative study, the accuracies of HRMRI and PET-CT were 83% and 70% respectively in the prediction of lymph node status of rectal cancer (24). A recent meta-analysis showed 77% sensitivity, 71% specificity and 8.3 diagnostic odds ratio (DOR) for N staging on MRI (21).

Imaging studies have shown a limited diagnostic accuracy using size criteria, but morphological criteria substantially improve this accuracy. Malignant nodes were found to have irregular outlines or to exhibit heterogeneous signal intensity on T2-weighted MRI (23,25,26). If either of these criteria was present, a sensitivity of 85% and a specificity of 97% were achieved for detecting nodal metastases in nodes ≥3 mm (26). 

There are some pitfalls in predicting LN status on MRI, leading to the false-positive results. First, as it has been discussed, the reactive LN swelling makes it difficult to be differentiated from involved nodes. Second, continuous (or sometimes discontinuous) tumour deposits could mimic the involved LN, resulting in overestimation of LN positivity. To avoid this kind of overestimation, direct continuity with the main tumour should be carefully assessed on multiplanar images. Differentiation between involved nodes and discontinuous extranodal tumour deposits (satellites) without residual nodal tissue has not been solved yet on microscopy, much less on imaging. Third, the postradiation effect is lying in wait to make the interpretation of MR imaging difficult. Edema of the perirectal fat tissue by radiation and postradiation fibrosis around the LN may result in false-positive results of LN status (23,27).

Preliminary experience using USPIO promises to improve this accuracy still further due to the ability to discriminate between malignant and non-malignant nodes based on the pattern of contrast uptake. Lahaye et al. reported that USPIO enhanced MR imaging had 93% sensitivity and 96% specificity in detecting lymph node metastases (28).

**Figure 2:**
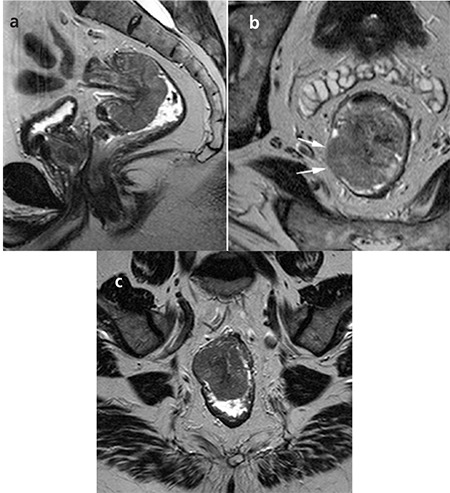
68-year-old man with pT2N1 rectal cancer. Two observersstaged the tumor as T2N0 in the preoperative MR imaging. a- T2-weighted sagittal MR image shows the polipoid tumor. b- T2-weighted para-axial MR images show the tumor with invasionof muscle layer but not extending beyond it. It was confirmed athistopathology. c- T2-weighted para-coronal MR image shows the tumor. Perirectalovoid lymph node measured 7x3.5 mm that has smooth bordersand homogeneous signal intensity was evaluated as benign. Buthistopathology revealed lymph node metastasis.

Our study using the size criteria for the detection of LN metastases had poor results (Figure 2). Among 30 patients, metastatic mesorectal lymph nodes (N1-2) were found in 12 patients. Predicting lymph node metastasis, the sensitivity was 58-58%, the specificity was 50-55%, the PPV was 43-46%, the NPV was 64-66%, and the area under the curve was 0.54-0.57. Statistically, there was poor correlation between pathologic and HRMRI node staging.

In the present study, the assessment was performed retrospectively on a per-patient basis on the pathology report and this is the main limitation of this study. It is difficult to indicate which of the LN was truly positive in patients with nodal involvement. The main problem in our study was poor prediction of lymph node status because of the reactive lymph nodes.

**CRM Involvement**

One of the major advantages of HRMRI is the visualisation of the mesorectal fascia. The identification of this important surgical landmark is the key to deciding whether neoadjuvant chemoradiotherapy is advisable. Several series have demonstrated the potential of MRI in predicting the distance between the tumour and this fascial plane (8,29,30).

Prediction of tumour-free CRM is important in the preoperative assessment of rectal cancer, and high-resolution rectal MRI is regarded as a superior preoperative imaging modality for this purpose. CRM involvement is the single most powerful predictor of local recurrence in rectal cancer, and consequently, assessment of the CRM, or mesorectal fascia, has become important in the assessment of patients (1,4,5). HRMRI has a sensitivity of 60-88% and a specificity of 73-100% for determining CRM status (11). A meta-analysis including nine studies and 529 patients reported that the sensitivity and specificity of MRI for detecting CRM involvement were 94% and 85% respectively (31). A recent meta-analysis revealed 77% sensitivity, 94% specificity and 56.1 diagnostic odds ratio (DOR) for predicting CRM involvement on MRI (21).

**Figure 3:**
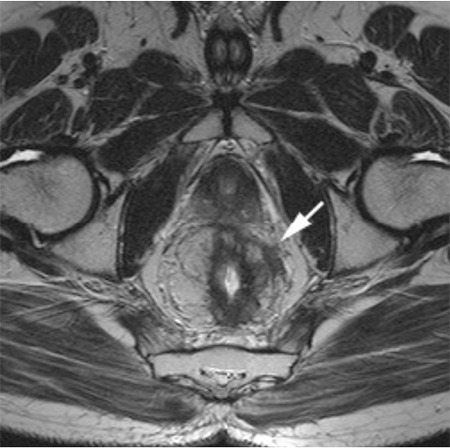
29-year-old man with pT4N1 rectal cancer. First observerstaged the tumor as T3N2, second observer staged as T4N2 in thepreoperative MR imaging. Two observers accurately predicted CRMinvolvement. T2-weighted para-axial MR image shows the tumor withmesorectal fascia invasion at the left side

Our study showed that MRI is able to predict those patients in whom the CRM is not involved, allowing them to proceed to surgery without the need for preoperative radiotherapy. Predicting CRM involvement, the PPV was 33-42%, the NPV was 79-81%, and the area under the curve was 0.63-0.65 (Figure 3). In 4 of 7 (41%) patients with affected margin, this had not been predicted by magnetic resonance imaging before the surgery. In four patients predicted by the first observer, and six patients by the second observer, although the local extent of tumour had been correctly documented compared with pathology, the distance to the mesorectal fascia had been overestimated. As the perirectal fat tissue is thinner at lower rectum, three of these patients having tumour at that localisation were overstaged by both observers. 

One of the important limitations of our study was the low number of patients. Moreover, since the study is a retrospective one, radiological-pathological correlation was conducted on the reports but not on macroscopic material, which was another important limitation. In the pathology reports, only the distance between the tumour and CRM was stated and the distance between the lymph node and the mesorectal facia we assessed in our patients was not stated in detail.

## CONCLUSION

As the conclusion, since the recent introduction of new treatment strategies for rectal cancer, there is a growing need for an accurate imaging tool to select patients preoperatively with different risks for recurrence so that treatment can be given according to the risks. Preoperative HRMRI does produce a reliable prediction of clear circumferential resection margins and provides valuable information in assessing whether patients can be proceeded to surgery without the need for preoperative radiotherapy. Our study shows that preoperative HRMRI provides good predictive data for extramural invasion but poor prediction of lymph node status because of the reactive lymph nodes and poor staging in CRM involvement.

## Figures and Tables

**Table 1:**
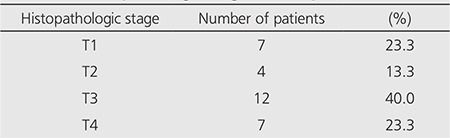
Histopathologic stages of the patients

**Table 2:**
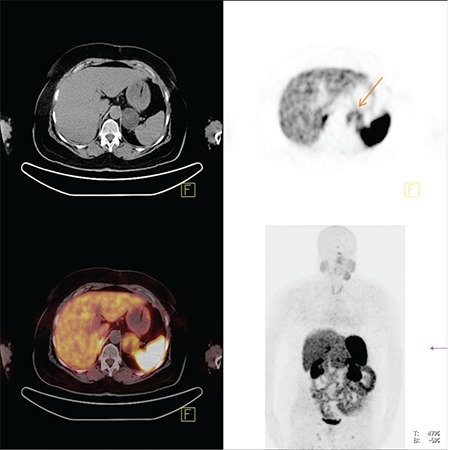
The efficacy of HRMR imaging in the prediction of extramural invasion

**Table 3:**
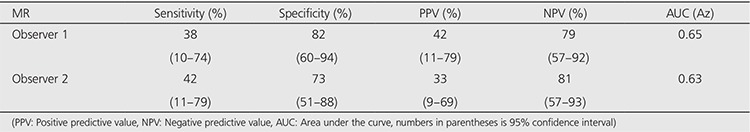
The efficacy of HRMR imaging in the prediction of CRM involvement

**Table 4:**
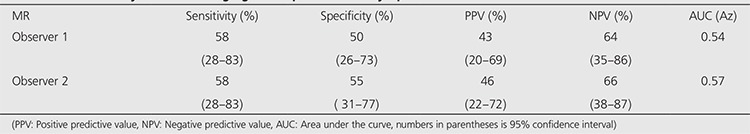
The efficacy of HRMR imaging in the prediction of lymph node metastases
